# Radiographic Features of COVID-19 in Children—A Systematic Review

**DOI:** 10.3390/children9111620

**Published:** 2022-10-25

**Authors:** Niamh Bergin, Niamh Moore, Shauna Doyle, Andrew England, Mark F. McEntee

**Affiliations:** Discipline of Medical Imaging & Radiation Therapy, University College Cork, T12 AK54 Cork, Ireland

**Keywords:** child, paediatric, infant, adolescent, chest X-ray, CXR, chest radiography, COVID-19, SARS-CoV-2, coronavirus

## Abstract

INTRODUCTION: The SARS-CoV-19 (COVID-19) pandemic has become a global problem but has affected the paediatric population less so than in adults. The clinical picture in paediatrics can be different to adults but nonetheless both groups have been subject to frequent imaging. The overall aim of this study was to comprehensively summarise the findings of the available literature describing the chest radiograph (CXR) findings of paediatric patients with confirmed COVID-19. The COVID-19 landscape is rapidly changing, new information is being constantly brought to light, it is therefore important to appraise clinicians and the wider scientific community on the radiographic features of COVID-19 in children. METHODS: Four databases, which included, PubMed; Medline; CINAHL; ScienceDirect were searched from the 30 November 2020 to the 5 March 2021. The review was conducted using the “Preferred Reporting Items for Systematic Reviews and Meta-Analysis, PRISMA” guidelines. Studies were included for (1) publications with full text available, (2) patients with confirmed COVID-19 diagnoses, (3) CXR imaging features of COVID-19 were reported, (4) the age of patients was 0–18 years, (5) studies were limited to human subjects and (6) a language restriction of English was placed on the search. Quality assessment of included articles used the National of Health Quality Assessment Tool for Case Series Studies. RESULTS: Eight studies met our criteria for inclusion in the review. All eight studies documented the number of CXRs obtained, along with the number of abnormal CXRs. Seven out of the eight studies noted greater than 50% of the CXRs taken were abnormal. Opacification was the number one feature that was recorded in all eight studies, followed by pleural effusion which was seen in six studies. Consolidation and peri-bronchial thickening features were both evident in four studies. Opacification was sub-divided into common types of opacities i.e., consolidation, ground glass opacities, interstitial, alveolar and hazy. Consolidation was reported in half of the studies followed by ground glass opacities and interstitial opacities which was seen in three out of the eight studies. CONCLUSION: This systematic review provides insight into the common COVID-19 features that are seen on CXRs in paediatric patients. Opacification was the most common feature reported, with consolidation, ground glass and interstitial opacities the top three opacifications seen. Peri-bronchial thickening is reported. in the paediatric population but this differs from the adult population and was not reported as a common radiographic finding typically seen in adults. ADVANCES IN KNOWLEDGE: This systematic review highlights the CXR appearances of paediatric patients with confirmed SARS-CoV-19, to gain insight into the disease pathophysiology and provide a comprehensive summary of the features for clinicians aiding optimal management.

## 1. Introduction

In Wuhan China, in December 2019, a group of patients presented with fever, cough, and pneumonia of an unknown source. Initial investigations found that this illness was the result of a novel coronavirus (SARS-CoV-2). The SARS-CoV-2 ‘coronavirus’ more commonly known as ‘COVID-19’, rapidly spread across the globe and led to COVID-19 being declared as a worldwide pandemic in March 2020. On the 20 May 2021, according to World Health Organisation (WHO), there had been 164,409,804 confirmed cases and 3,409,220 deaths [1].

Many published studies stated that the individuals most frequently affected during the Pandemic were adults over 60 years of age, there has also been many COVID-19 cases seen in paediatrics, including infants, children and young adults. In 2021, the WHO, stated that children and young adults would face many challenges based on their phase of life and from both the COVID-19 disease and the measures created to contain the disease [1]. Children and young people typically comprise only 1–2% of cases of COVID-19 worldwide [2]. COVID-19 has appeared to have a minimal effect on children, with reports of only a low number of symptomatic and severe cases compared to adults [3,4]. In the majority children will be symptomatic for only a few of days and symptoms will resolve naturally. Although children tend to have milder symptoms, like all humans they can be agents for transmission and are therefore important to identify promptly.

The early detection and treatment of individuals affected by COVID-19 is critical. Lung imaging plays an important role and to date the most frequently used imaging modalities are chest radiography (CXR) and computed tomography (CT) [5]. CT has become extremely valuable in the screening, diagnosis and aftercare of patients with COVID-19 and provides medical practitioners with important diagnostic information. Radiological studies are less frequently requested in children due to the overall lower rates of infection [6] and the generally milder nature of the disease. COVID-19 features on imaging appear to be changeable with age and there are possible distinct features in infants, children and adolescents. Appearances of COVID-19 on lung imaging in adults have been previously documented in the literature, but by comparison documentation of the lung disease patterns of COVID-19 in a paediatric population remains less clear [7]. Paediatric clinicians also face additional challenges when attempting to differentiate early stages of COVID-19 infection from other types of viral lower respiratory tract infections. In addition, a small number of COVID-19 positive children will go on to develop Paediatric Inflammatory Multisystem Syndrome (PIMS). PIMS can present with a range of symptoms and evaluation in severe cases may include imaging.

Although, imaging is commonly used in the management of adults with COVID-19, radiology is likely not to be routinely required in paediatric cases, especially if the child is asymptomatic [4]. A child is more sensitive to radiation exposure; therefore, routine use of CT is not recommended, which makes a distinct difference in their radiological work-up in contrast to adults [8]. Also, the American College of Radiology [9] does not recommend medical imaging examinations as a formal method of COVID-19 diagnosis, and confirmation of COVID-19 by PCR testing is key even if the radiological appearances are highly suggestive of COVID-19 [6].

Referral for imaging is part of the management plan for clinicians [5]. It is vitally important that clinicians of all specialties recognise the appearances of COVID-19 on radiographic images, especially when the clinicians are suspecting something other than COVID-19.

The overall aim of this study was to undertake a comprehensive evaluation of the findings of published literature which have described the CXR features in children with confirmed COVID-19. To achieve the aim, the research team undertook a systematic review of the published literature across different databases to identify (1) the number of children with normal chest radiography, (2) the incidence of different radiographic (CXR) abnormalities reported in PCR-confirmed paediatric SARS-CoV-2 cases. Considering the low number of children which will require imaging it is important to provide up to date information regarding the appearances of COVID-19 on chest radiography. This in turn will help improve knowledge of COVID-19 and improve diagnosis and management.

## 2. Materials and Methods

### 2.1. Search Strategy

**Figure 1:**
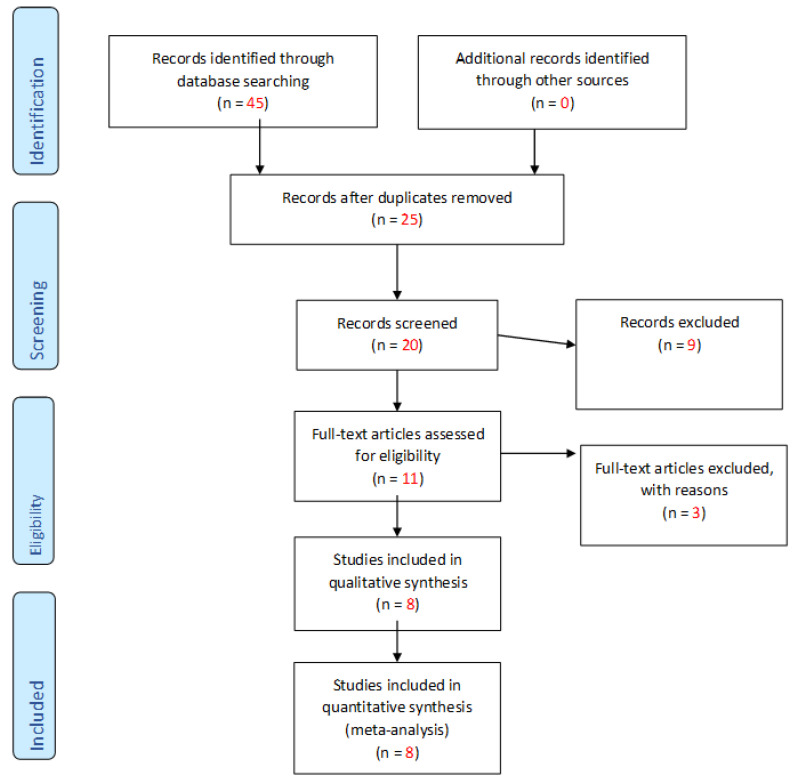
Diagram of retrieval of studies, using “Preferred Reporting Items for Systematic reviews and Meta-Analyses, PRISMA”.

An extensive electronic search was conducted in the following databases PubMed, MEDLINE, ScienceDirect and CINAHL. All procedures in the review were executed in accordance with the “Preferred Reporting Items for Systematic reviews and Meta-Analyses, PRISMA,” [10] guidelines (Figure 1). A methodological search of literature was undertaken from the 30 November 2020 to the 5 March 2021. An initial search of the literature was performed on 17 February 2021 and a second ‘repeat’ search was run on 5 March 2021. As this continues to be an ever-evolving field there is a rapid number of studies being published every day. The above systematic search was reviewed by a second researcher to ensure transparency throughout the searching process.

### 2.2. Inclusion and Exclusion Criteria

Studies were selected for potential inclusion based on full text analysis of the title, abstract and keywords. Criteria for inclusion included all studies which described or investigated chest radiography findings of COVID-19 confirmed infections in children. Studies were eligible for inclusion if (1) publications were available in full text, (2) contained patients who had confirmed COVID-19 diagnoses, (3) CXR imaging features of COVID-19 were included in the publication, (4) the age of patients was between 0–18 years, (5) studies were limited only to humans and (6) articles had to have been published in English.

Studies were excluded if they were (1) letters, theses, books, editorials or posters, (2) studies on the adult population, (3) any studies reporting on a mixed paediatric/adult cohort and specifically where imaging results for the paediatric cohort could not be extracted, (4) lack of clinical data presented, (5) no PCR-confirmation of COVID-19 infection and (6) duplicate studies.

### 2.3. Risk of Bias

Quality assessment of the included literature was determined using the National Institutes of Health (NIH) Quality Assessment Tool for Case Series Studies [11], from this general quality ratings were categorized as poor, fair, or good (Table 1). Two reviewers independently graded the quality of the selected articles. Any disagreements between reviewers were resolved through discussion, and if necessary, a third reviewer was introduced to make the final decision.

### 2.4. Data Extraction

Data extraction was performed independently by the primary reviewer using a data extraction tool adapted from the Cochrane Collaboration [17]. This form has been developed by adopting and customizing the “Data collection form for intervention review-RCT’s and non-RCT’s” of the Cochrane Collaboration. All information was collected and transcribed onto an Excel spreadsheet. Data that was inserted into the Excel spreadsheet was then reviewed separately by the second reviewer. As previously stated, if any disagreements arose, they were resolved by discussion, and if necessary via a third reviewer.

## 3. Results

### 3.1. Selection of Articles

Following the initial search, a total of 45 papers were identified from the four databases previously mentioned. After removing 25 duplicates, a total of 20 publications were included for the screening process. Manual screening of the title and abstract of these 20 papers resulted in 11 papers being included for the full-text review. From the full-text review, a total of eight papers met the inclusion criteria and were included in this systematic review. The PRISMA flowchart representing the search results is illustrated in Figure 1. The two independent reviewers agreed with the study selection and no discrepancies were found during the research process

### 3.2. Methodological Quality

The quality assessment resulted in four of the articles receiving an overall scoring of “good”, with the remaining four receiving a score of “fair”. Further details on the methodological quality assessment of the included studies is presented in Table 1. Improvements in the reporting of the statistical analysis of the studies included would have increased the quality grading of the four ‘fair’ rated studies.

Table 2 provides a summary of the key features of the studies included. All eight included studies were retrospective in enrolment. The enrolment period for the studies commenced between January 2020 and April 2020 and was completed between February 2020 and May 2020. The country of origin varied in six out of eight studies and were predominantly in the US, China and Europe. Six out of the eight studies were conducted in a single centre and the other two studies were multi-centre. The mechanism of selection of the participants was consecutive for two studies, the remaining studies (n = 6) failed to describe the selection mechanism.

Table 3 presents the patient demographics from the eight selected papers. The number of children studied in each of the papers varied greatly and not every child required CXR. In total, there were 762 children included in this systematic review of which 367 required a CXR, (209/367 [57%] were abnormal). All reports considered both male and female patients, but the median age of all patients varied but was still within the inclusion criteria of 0–18 years. All eight papers documented that all patients received a positive PCR test, but it is unclear which of these had a diagnosis of COVID-19 on CXR or with PCR testing. Seven out of eight studies stated the number of patients that were symptomatic or asymptomatic. But only three of the papers stated whether their patients had comorbidities (n = 166) before contracting coronavirus. It would be important to be aware of comorbidities as the related radiological appearances could be misread or misdiagnosed as COVID-19. Finally, all eight papers did document the number of normal CXR (n = 158) that were obtained, and they also stated the number of abnormal CXRs (n = 209), but the eight papers did not document specific details with regards to the sensitivity or the specificity of the CXR against PCR testing.

### 3.3. Chest Radiography Appearances of COVID-19 in Children:

The CXR COVID-19 appearances from the eight papers are shown in Table 4 below. Opacification was present in all eight studies, followed by pleural effusion which was present in six studies. Consolidation and peri-bronchial thickening features was found in four out of the eight studies. Less common features such as cardiomegaly, congestive heart failure, ARDS, pneumothorax, atelectasis, and mediastinal widening were present in one—two studies. The location of the features was documented in two out of the eight papers, with one study seeing 4% unilateral and 4% bilateral and the other study seeing 25% unilateral and 20% bilateral. Distribution of the features was documented in seven studies, six of the these showed that the distribution is predominantly in the perihilar or central regions of the lungs.

Table 5 sets out the common COVID-19 features. All eight studies reported opacifications. This was sub-divided into common types of opacities i.e., consolidation, ground glass opacities, interstitial, alveolar and hazy. Consolidation was the most common and was evident in half of the studies, followed by both glass opacities and interstitial opacities was seen in three out of the eight studies.

## 4. Discussion

This review of the literature utilizing a systematic methodology has provided a comprehensive evaluation of the published literature to date which have considered the CXR features of COVID-19 in children. This systematic review includes children from new-borns to adulthood (18 years old), with positive PCR testing confirming a COVID-19 infection.

The study enrolment on all eight studies, was retrospective which introduces a lower risk of bias to this systematic review. But all studies examined were at the initial stages of the pandemic between January–May 2020 and covered a short time of between 0.5–2.5 months. The limited number of publications available for inclusion and supports the initial findings that there are only a few studies carried out on the use of CXR and its related imaging appearances of COVID-19 in children in contrast to those available for adults.

It is important to note that not all children that test positive will require or should undergo a CXR or CT examination. In the systematic review, all eight studies stated the number of children that tested positive and the number of children that required CXR. In two of the eight studies less than half of the children required CXR whereas six out of the eight studies noted that greater than half or all the children required CXR. However, due to the lack of information regarding the severity of the symptoms and clinical status of the children (comorbidities), it is difficult to determine the justification for the high percentage of children requiring CXR.

Seven out of the eight studies documented whether the child was symptomatic or asymptomatic with six of these reporting that the children were symptomatic, only Biko et al. [12] reported that most of the children included in their study were asymptomatic. Given the lack of detailed information, it is difficult to determine whether and if so why asymptomatic paediatric patients underwent CXR.

Six case studies showed that where children required imaging, CXR was the preferred method. This is in line with a number of case studies and guidelines [9,11] where it has been cited that in paediatric patients, it is vital to use the lowest radiation doses possible for a diagnosis which would be in accordance with the ALARA principle. However, it should also be noted that imaging should only be undertaken in specific circumstances if symptoms worsen or are persistent.

Of the eight studies reviewed, only five studies stated their patients’ comorbidity status. Three studies reported comorbidities in their patients whereas two studies [6,16] reported no comorbidities with any of their patients. Of the studies that reported comorbidities, Biko et al. [12] and Blumfield et al. [13] highlighted that more than half of their patients had comorbidities before acquiring a COVID-19 infection, whereas the study by Caro-Dominquez et al. [14] reported a third of their patients had comorbidities before becoming infected. The type of comorbidity a patient may have prior to contracting COVID-19 may dictate the clinical presentation of the patient, which in turn could influence the radiological appearances on their CXR and may lead to misinterpretation or misdiagnoses of COVID-19. Given the potential for misdiagnosis, it is important that neither CXR nor chest CT is used to screen for COVID-19 or as a first-line of investigation to diagnose symptomatic COVID-19 [6]. However, in children presenting with moderate or severe symptoms and those with underlying risk factors, it has been reported that CXR can be useful in establishing an imaging baseline as well as assessing for alternative diagnosis [8].

Overall, seven out of the eight studies noted greater than 50% of the CXRs taken were abnormal however the lack of information regarding the severity of the symptoms makes it problematic when determining the reason behind the high numbers of abnormal CXRs.

In this review, all eight studies had one common clinical finding, which was the presence of opacifications. Both peri-bronchial thickening and pleural effusion were reported in six out of the eight studies, where consolidation was reported in four out of the eight studies. Finally, there were a number of infrequent features reported in paediatric patients including: atelectasis which was reported in two studies and cardiomegaly, congestive heart failure, ARDS, pneumothorax and mediastinal widening which were reported in one study each.

Study reviewers further assessed features that may be specific to COVID-19: including peri-bronchial thickening, ground glass opacities, consolidation [6] as well assessing CXRs for the distribution and type of pulmonary opacities, i.e., interstitial, hazy and consolidation [13]. All eight studies reported opacities on their CXRs, these opacities included consolidation. This is similar to adults where the most common radiographic features are airspace opacities, which are most commonly described as consolidation and less commonly as ground glass opacities [15,16]. Peri-bronchial thickening was a feature in three out of the eight studies in this review. This contrasts to the common radiographic findings for adults where peri-bronchial thickening was uncommon and nonspecific for COVID-19 [6,14].

## 5. Limitations

Firstly, only a small number of case studies were included in this systematic review. Furthermore, some of these studies were limited in terms of sample size. This could have potentially introduced bias. Secondly, the review was limited to publications written in English.

All the studies examined were carried out during the initial months of the pandemic, when there were many unknowns. It is possible that more recent studies on COVID-19 may provide additional findings. It would have been useful if imaging appearances could have been correlated against the time of presentation/day of hospitalization. It is highly probable that the time since the onset of infection would influence the frequency and severity of the imaging findings. This would, therefore, affect the results of this systematic review and readers should consider this context when interpreting our findings. Further sub-analysis of data could also be introduced, and this could consider the coronavirus strain togetherwith differences in the sex and age of the child and the presence of comorbidities.

Lastly, the reviewer noted a lack of detailed data and information regarding the patients’ ages, symptoms, including symptom severity. In the absence of this information, the reviewer was unable to compare potential features associated with certain age groups specific and include symptoms that the patients presented with. Furthermore, the reviewer was not able to understand and develop patterns between the number of patients who were positive for a COVID-19 infection and who had abnormal CXRs and the linkage of this with the chest radiographic features and common COVID-19 features.

## 6. Conclusions

This systematic review provides a detailed evaluation of the currently available literature on the CXR appearances of COVID-19 in paediatrics. This review demonstrated seven studies where greater than 50% of their cohort had abnormal CXRs. Opacification was the number one feature reported in the studies, with consolidation, ground glass and interstitial opacities the main opacifications reported. Peri-bronchial thickening is one radiographic finding seen in the paediatric population but this is not typically seen in adults. Given the time elapsed since the first reported COVID-19 case there will be further experiences and data on the effects on children. Work is needed to identify any specific patient characteristics that may influence disease severity and progression, such factors may include sex, age and existing comorbidities.

## Figures and Tables

**Table 1:** Summary of the quality ratings, according to the National Institute of Health (NIH) Quality Assessment Tool for Case Studies, of the included studies.

*Article*:	Bayramoglu et al. [6]	Biko et al. [12]	Blumfield et al. [13]	Caro-Dominguez et al. [14]	Hameed et al. [15]	Lu et al. [16]	Oterino Serrano et al. [8]	Palabiyik et al. [4]
Q1: Was the study question or objective clearly stated?	YES	YES	YES	YES	YES	YES	YES	YES
Q2: Was the study population clearly and fully described, including a case definition?	YES	YES	YES	YES	YES	YES	YES	YES
Q3: Were the cases consecutive?	YES	YES	NR	NR	NR	NR	NR	NR
Q4: Were the subjects comparable?	NO	NO	NO	NO	NO	NO	NO	NO
Q5: Was the intervention (i.e., imaging modality) clearly described?	YES	YES	YES	YES	NO	YES	YES	YES
Q6: Were the outcome measures clearly defined, valid, reliable, and implemented consistently across all study participants?	YES	YES	YES	YES	NO	YES	YES	YES
Q7: Was the length of follow-up adequate?	NA	NA	NA	NA	NA	NA	NA	NA
Q8: Were the statistical methods well-described?	YES	YES	NO	NO	NO	NR	YES	YES
Q9: Were the results well-described?	YES	YES	YES	YES	YES	YES	YES	YES
Quality Rating: Reviewer 1	GOOD	GOOD	FAIR	FAIR	FAIR	FAIR	GOOD	GOOD
Quality Rating: Reviewer 2	GOOD	GOOD	FAIR	FAIR	FAIR	FAIR	GOOD	GOOD

NA—not applicable; NR—not reported.

**Table 2:** Summary of studies characteristics.

Article:	Bayramoglu et al. [6]	Biko et al. [12]	Blumfield et al. [13]	Caro-Dominguez et al. [14]	Hameed et al. [15]	Lu et al. [16]	Oterino Serrano et al. [8]	Palabiyik et al. [4]
Patient enrolment:	Retrospective	Retrospective	Retrospective	Retrospective	Retrospective	Retrospective	Retrospective	Retrospective
Country:	Istanbul	Philadelphia	New York (Bronx)	The Netherlands European Society of Paediatric Radiology	London	China	Spain	Istanbul
Enrolment beginning:	10 March 2020	17 March 2020	25 February 2020	12 March 2020	14 April 2020	22 January 2020	13 March 2020	11 March 2020
Enrolment ending:	31 May 2020	21 May 2020	1 May 2020	8 April 2020	9 May 2020	9 February 2020	6 April 2020	20 April 2020
Type of Study:	Single Centre	Multi Centre	Single Centre	Multi-centre	Single Centre	Single Centre	Single Centre	Single Centre
Consecutive/Random selection:	Consecutive	Consecutive	Not Reported	Not Reported	Not Reported	Not Reported	Not Reported	Not Reported

**Table 3:** Summary of patient demographics.

Article:	Bayramoglu et al. [6]	Biko et al. [12]	Blumfield et al. [13]	Caro-Dominguez et al. [14]	Hameed et al. [15]	Lu et al. [16]	Oterino Serrano et al. [8]	Palabiyik et al. [4]
Number of patients diagnosed with COVID-19	74	313	19	91	35	9	44	177
Number of patients requiring CXR	69	51	19	81	35	9	44	59
Total number (median age, years)								
Male:	36 (11)	164 (6.6)	10 (8)	49 (6.1)	27 (11)	5 (7.8)	29 (6.6)	34 (9)
Female:	38 (12)	149 (9.4)	9 (8)	42 (6.1)	8 (11)	4 (7.8)	15 (6.6)	25 (9)
Symptomatic	NR	92	19	85	35	8	44	59
Asymptomatic		221		6		1		
Comorbidities:	0	41(74.5%)	12 (63.2%)	30 (33%)	NR	0 (0%)	NR	NR
Number of Normal CXR:	56 (81.1%)	34 (66.6%)	1 (5.2%)	10 (12.3%)	16 (45.7%)	5 (55.6%)	4 (9%)	32 (54.2%)
Number of Abnormal CXR:	13 (18.8%)	17 (33.3%)	18 (94.8%)	71 (87.7%)	19 (54.3%)	4 (44.4%)	40 (90.9%)	27 (45.8%)
Received PCR Test:	YES	YES	YES	YES	YES	YES	YES	YES
Sensitivity:	NR	NR	NR	NR	NR	NR	NR	NR
Specificity:	NR	NR	NR	NR	NR	NR	NR	NR

**Table 4:** Summary of chest radiographic features.

Article:	Bayramoglu et al. [6]	Biko et al. [12]	Blumfield et al. [13]	Caro-Dominguez et al. [14]	Hameed et al. [15]	Lu et al. [16]	Oterino Serrano et al. [8]	Palabiyik et al. [4]
Consolidation:			13 (68.4%)	28.3 (35%)	5 (14.2%)		8 (18.1%)	
Opacifications:	6 (8.6%)	30 (58.8%)	15 (78.9%)	28.4 (35%)	11 (31.4%)	4 (44.4%)	32 (72.7%)	27 (45.8%)
Peri bronchial Thickening:	7 (10.1%)			47 (58%)	12 (34.3%)		38 (86.3%)	
Pleural effusion:	1 (1.4%)	5 (9.8%)	4 (21%)	6 (7.4%)	4 (11.4%)		4 (9.1%)	
Cardiomegaly:			7 (36.8%)					
Congestive heart failure:			7 (36.8%)					
ARDS:			2 (10.5%)					
Pneumothorax:				2 (2.4%)				
Atelectasis:				2 (2.4%)	7 (20%)			
Mediastinal widening:							2 (4.5%)	
Location of Features:		NR	NR	NR	NR	NR	NR	
Unilateral:	3 (4.4%)							15 (25.4%)
Bilateral:	3 (4.4%)							12 (20.3%)
Distribution of features:				NR				
Perihilar (central):	3 (4.4%)	2 (6.6%)	11 (73.3%)		11 (31.4%)	4 (44.4%)	17 (38.6%)	
Peripheral:	3 (4.4%)	3 (10%)	1 (6.6%)				5 (11.4%)	31 (22%)
Diffused:		14 (46.6%)	5 (33.3%)				37 (84%)	16 (27.1%)
Lower lobes:			9 (60%)					
Scattered:		3 (10%)						
Not well-defined:		2 (6.6%)						

**Table 5:** Summary of common COVID-19 features.

Article:	Bayramoglu et al. [6]	Biko et al. [12]	Blumfield et al. [13]	Caro-Dominguez et al. [14]	Hameed et al. [15]	Lu et al. [16]	Oterino Serrano et al. [8]	Palabiyik et al. [4]
Peri-bronchial Thickening:	7 (10.1%)			47 (58%)	12 (34.3%)			
Opacities:	5 (7.2%)	30 (58.8%)	15 (78.9%)	56 (70.4%)	16 (45.7%)	4 (44.4%)	40 (90.9%)	27 (45.8%)
Ground Glass Opacities	ü 5 (7.2%)			ü 15 (18.9%)			ü 22 (50.0%)	
Interstitial		ü 16 (31.4%)	ü 6 (31.6%)	ü 12 (15.1%)				
Alveolar		ü 14 (27.4%)						
Consolidation			ü 13 (68.4%)	ü 28 (35.2%)	ü 5 (14.3%		ü 8 (18.2%)	
Hazy			ü 8 (42.1%)					
